# Relapse Burden of Severe Acute Malnutrition Post‐Recovery Using SPHERE Performance Indicators: A Systematic Review and Meta‐Analysis

**DOI:** 10.1002/hsr2.71528

**Published:** 2025-11-19

**Authors:** Fassikaw Kebede Bizuneh, Birtukan Gizachew, Tsehay Kebede, Abebe Fenta, Desalegn Nazi, Sefineh Fenta, Worku Misganaw

**Affiliations:** ^1^ College of Medicine and Health Science Debre Markos University Debre Marqos Ethiopia; ^2^ College of Health Science Woldia University Weldiya Ethiopia; ^3^ College of Social Science Bahir Dar University Bahir Dar Ethiopia; ^4^ College of Health Science Assosa University Asosa Ethiopia

**Keywords:** acute malnutrition, children, Ethiopia, relapse, treatment recovery

## Abstract

**Background:**

In Ethiopia, factors including persistent regional conflicts, comorbidities, and caregivers' self‐discharge (ranging from 2.5% to 36.5%) from treatment centers increase the risk of readmission. This study aimed to estimate the relapse burden of SAM utilizing SPHERE performance indicators through a systematic review and meta‐analysis (SRMA).

**Methods:**

Following the PRISMA guideline, we retrieved 1276 published articles from PubMed, Scopus, ScienceDirect, Web of Science, African Journals Online, and Google Scholar. Article quality was assessed using the JBI checklist. The pooled relapse burden was estimated using weighted inverse variance random‐effects meta‐regression. Heterogeneity was evaluated using Cochran's Q test and I² statistics. Subgroup analyzes and sensitivity tests were conducted to explore potential publication bias.

**Result:**

Eight studies comprising 4,878 participants were included, of which 958 cases were readmitted for SAM re‐treatment. The male‐to‐female ratio was 47.2% to 52.8%. The pooled national relapse burden was 19.4% (95% CI: 10.69 to 28.78, I² = 98.78%). Aggregating treatment outcome indicators using SPHERE performance standards yielded recovery, attrition, and death rates of 79.17%, 4.15%, and 5.84%, respectively. Subgroup analysis indicated a higher relapse burden in the Amhara region (RB = 26.9%) compared to the Southern region (RB = 10.52%). Random‐effects meta‐regression identified MUAC ≥ 125 mm as a discharge criterion (Pooled HR 3.6) and household food insecurity (Pooled HR = 6.33) as significant predictors for SAM relapse.

**Conclusion:**

This review found that nearly one in every five children under five is readmitted for SAM re‐treatment. The relapse burden and loss to follow‐up rates exceeded the expected national‐level SPHERE performance indicators. Therefore, the use of MUAC indices for recovery discharge criteria should be reconsidered and re‐standardized. Comprehensive nutrition education, household food aid, and continued follow‐up are strongly recommended for children who have been treated and declared cured of severe acute malnutrition.

**Trial Registration:**

PROSPERO CRD42024520816.

## 1 Introduction

Malnutrition is a leading cause of child morbidity and mortality worldwide, responsible for 60% of the 10.9 million annual deaths among children [1, 2]. It occurs when food intake does not meet the dietary needs of vulnerable children [3]. Malnutrition results from a complex interplay of nutritional disorders, including underweight (low weight‐for‐age, Z‐score ≤ −2), wasting (low weight‐for‐height, Z‐score ≤ −2), or stunting (low height‐for‐age, Z‐score ≤ −2), coupled with micronutrient deficiencies and comorbidities [4]. It is a critical nutrient deficiency associated with long‐term nutritional malabsorption [5]. The 2022 Global Malnutrition Report indicated that 22% and 7% of children under five were stunted and wasted, respectively; countries in Asia and Africa accounted for ≥ 90% of this burden [6]. Although SAM can affect all age groups, infants ≤ 24 months are at higher risk [4, 6], with the peak risk age being 6–18 months [2]. In 2019, an estimated 52 million children under five had SAM, and 2.9 million were admitted for treatment [7]. According to a 2023 joint report by UNICEF, WHO, and the World Bank, 16.4 million children under five (2.4%) suffered from SAM [8], of which 2.2 million were from Sub‐Saharan Africa (SSA) [1].

Children with SAM often endure long‐lasting repercussions, facing a 5 to 20 times higher mortality risk compared to well‐nourished children [6, 9]. Case fatality rates for SAM have decreased from 16% to 8% following the implementation of WHO outpatient treatment protocols [6]. However, in Ethiopia, at least 25,000 children are admitted for SAM treatment annually [10, 11]. Premature discharge before achieving target weight gain can heighten the risks of impaired physical function, disability, and readmission for SAM re‐treatment [12]. Children who survive SAM are more likely to perform poorly in school and experience significantly increased mortality [4, 13]. Previous studies in low‐ and middle‐income countries (LMICs) [1, 14, 15] have reported that poor feeding practices, limited healthcare access, and socioeconomic disparities among caregivers aggravate post‐recovery relapse. In Ethiopia [16], 10 high‐impact, nutrition‐specific interventions have been identified for malnutrition treatment [17], including 90% coverage for the aggressive management of SAM and moderate acute malnutrition (MAM), with a 2‐week follow‐up [3, 14].

WHO management protocols prioritize high program coverage, including outpatient management with ready‐to‐use therapeutic food (RUTF) for children with appetite and clinical stability (‘uncomplicated’ SAM), and inpatient treatment for complicated SAM cases [16]. Children with uncomplicated SAM (weight‐for‐height below ‐3 SD and/or MUAC < 11.5 cm and/or bilateral edema) are treated in stabilizing centers [1]. Those with a WHZ between −2 and −3 and/or MUAC between 11.5 and 12.5 cm are managed in community‐based programs using special therapeutic foods [1, 16]. Children are discharged when their weight‐for‐height/length is ≥ −2 Z‐score with no edema for 2 weeks, or if MUAC is ≥ 12.5 cm with no edema for the same period [1, 18]. However, recent findings [14, 15, 19, 20, 21, 22] suggest that poor adherence by healthcare providers to WHO treatment guidelines, lack of follow‐up, inadequate post‐discharge nutrition counseling, and subpar referral systems increase the risk of relapse [23]. Most SAM treatment centers lack baseline benchmarks for the universal performance of minimum SPHERE humanitarian charters [2].

SPHERE performance indicators are used to evaluate SAM treatment outcomes in resource‐limited healthcare facilities. These include minimum acceptance criteria for recovery (≥ 75%), mortality (≤ 10%), loss to follow‐up (≤ 10%), transfer out (≤ 15%), and relapse rate (≤ 5%) [2, 3]. However, a prospective cohort study in Mali reported readmission rates ranging from 1.7% to 26.1% [24]. Rates vary across developed and developing countries, including 7.4% in Pakistan [25] and 15.4% in Burkina Faso [15]. In India, 18‐month post‐discharge relapse rates varied from 9.1% at 3 months to 12.0% at 6 months [25]. In Ethiopia, approximately 7.6% to 20.6% of caregivers self‐discharge from treatment centers [26, 27, 28], which, combined with limited healthcare access and regional conflict [29], increases the risk of post‐recovery relapse. Therefore, this study aimed to estimate the relapse burden of SAM and identify predictors using SPHERE performance indicators through a systematic review and meta‐analysis.

## 2 Methods

### 2.1 Study Setting and Methods

This systematic review and meta‐analysis (SRMA) includes articles published exclusively in Ethiopia. Ethiopia is a Federal Democratic Republic with a government structure comprising nine regional states (Afar, Amhara, Benishangul‐Gumuz, Gambella, Harari, Oromia, Somali, Southern Nations, Nationalities, and Peoples' Region, and Tigray) and two city administrations (Addis Ababa and Dire Dawa) [30]. The country spans 1,100,000 km² and is administratively divided into zones, districts, and kebeles (the smallest administrative units). With a population of approximately 112 million people, Ethiopia is Africa's second‐most populous nation, with 56,010,000 females and 56,069,000 males reported in 2019 [31, 32].

### 2.2 Protocol Registration

The study protocol for this systematic review and meta‐analysis was registered on PROSPERO (registration number CRD42024520816). The review included all observational study designs (cross‐sectional, case‐control, survey, retrospective, and prospective cohort studies) focusing on the burden of acute malnutrition. The Preferred Reporting Items for Systematic Reviews and Meta‐Analyzes (PRISMA‐2020) guideline was utilized **(S1 PRISMA 2020 Checklist)** [33].

### 2.3 Data Searching Strategy

An extensive article search was conducted across six international electronic databases: PubMed (*N* = 573), Scopus (*N* = 285), Web of Science (*N* = 246), Google Scholar (*N* = 39), and Medline (*N* = 73), yielding 1276 published articles. The search focused on English‐language articles addressing the relapse burden of acute malnutrition post‐discharge. Boolean terms (AND, OR) and MeSH terms were used. The PubMed search strategy included: “Recurrence”(Mesh Terms) OR relapse (Text Word) AND burden (All Fields) AND post (All Fields) AND (“therapy”[Subheading] OR “therapeutics” [Mesh Terms] OR treatment [Text Word]) AND “patient discharge” (Mesh Terms) OR discharge (Text Word) AND acute (All Fields) AND (“malnutrition” [Mesh Terms] OR malnutrition [Text Word]) AND under (All Fields) AND five (All Fields) AND “child” (Mesh Terms) OR children (Text Word). Article eligibility was assessed using the PRISMA checklist.

### 2.4 Study Population and Inclusion Criteria

This SRMA focused on all observational study designs examining the relapse burden of acute malnutrition in children post‐recovery from discharge feeding centers. Only English‐language publications were included. The PICO (Population, Intervention, Comparison, and Outcome) framework was used to structure the research question:

- **Population (P):** Children under five diagnosed with severe acute malnutrition.
- **Intervention (I):** Children with SAM who experienced relapse post‐treatment.
- **Comparison (C):** Children under five without relapse.
- **Outcome (O):** Relapse.

Participants included both male and female children aged 6–59 months. The study design encompassed all observational types.

### 2.5 Exclusion Criteria

Articles lacking full‐text abstracts, publication years, author names, case reports, and inaccessible full texts were excluded.

### 2.6 Outcome Ascertainment

This SRMA had two main outcomes: (1) estimating the proportion of relapse burden of acute malnutrition post‐treatment, calculated as the number of relapsed cases divided by the total participants, multiplied by 100 to obtain the effect size using STATA version 17; and (2) identifying significant predictors for relapse by pooling aggregated estimates of risk factors using weighted inverse variance random‐effects meta‐regression.

### 2.7 Operational Definitions

- **Severe acute malnutrition (SAM):** Defined as WHZ < −3 or MUAC < 115 mm, with bilateral pitting edema, loss of appetite, and inability to take medications or feed orally. Treatment in a stabilizing center involves nasogastric intubation and parenteral antibiotics across three phases until oral intake is tolerated [34].
- **Moderate acute malnutrition (MAM):** Defined as WHZ between −2 and −3 or MUAC between 115 mm and 125 mm [35].
- **Relapse/readmission:** Defined as a child having multiple admissions for SAM, specifically when WHZ is between −2 and −3 or MUAC is 115−125 mm [36].

### 2.8 Article Quality Assessment and Appraisal

The methodological quality of included publications was assessed independently by two reviewers (FKB and GTB) using the Joanna Briggs Institute (JBI) checklist for observational studies **(S2 JBI Checklist)** [37]. Criteria included appropriate statistical analysis, strategies for incomplete follow‐up, sufficient follow‐up time, valid and reliable outcomes, participants free of the outcome at baseline, identification of confounding factors, and strategies to handle missing data. Questions meeting these criteria were scored “1,” otherwise “0.” Studies scoring ≥ 50% were considered low‐risk/high‐quality.

### 2.9 Data Extraction and Screening Process

Two authors (FKB and TKB) screened article titles and abstracts against inclusion and exclusion criteria. Two reviewers (BG and WMK) extracted relevant data from October 16 to November 21, 2024. Disagreements were resolved through discussion with a third author (DNJ). Data extracted included first author, publication year, study region, design, setting, sample size, outcome, response rate, mortality incidence, and predictors' effect size (AHR) into a Microsoft Excel spreadsheet.

### 2.10 Handling of Missing Data

Missing data were addressed using complete case analysis. As missing values constituted less than 5%, single or multiple imputations was not performed [38].

### 2.11 Software and Statistical Analysis

Citations were managed using EndNote version 8.1, with duplicates removed during screening. Data were extracted using Microsoft Excel (Meta‐XL) version 5.3 and exported to STATA version 17 for analysis [39]. The pooled relapse burden and predictors were estimated using weighted inverse variance random‐effects meta‐regression with DerSimonian‐Laird model weights [40, 41]. Descriptive statistics were presented in tables and funnel plots.

### 2.12 Publication Bias and Sensitivity Analysis

Subgroup analysis was conducted to address heterogeneity. Heterogeneity was assessed using Cochran's Q‐test and I² statistics. Publication bias was evaluated qualitatively via funnel plot inspection and quantitatively using Begg's and Egger's regression tests. A leave‐one‐out sensitivity analysis was performed to assess the influence of individual studies on the pooled estimate.

## 3 Result

### 3.1 Screening of Included Studies

**Figure 1:**
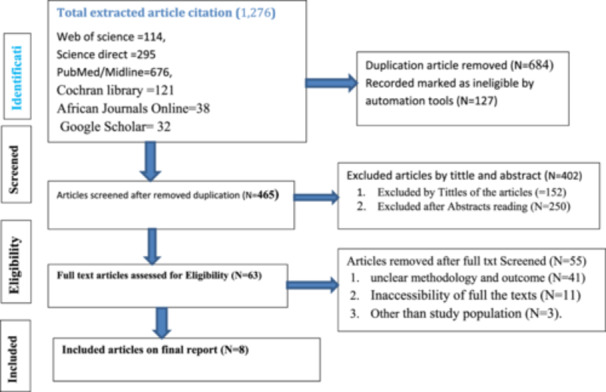
PRISMA 2020 flow diagram of article selection for systemic review and meta‐analysis.

The initial search yielded 1276 articles. After removing 811 duplicates, 465 articles underwent title and abstract review, leading to the exclusion of 402 articles that did not meet inclusion criteria. Sixty‐Five publications were assessed for full‐text review, and 55 were excluded due to unclear methodology or outcomes (N = 41), inaccessible full texts (*N* = 11), or irrelevant study population (*N* = 3). Ultimately, eight studies [9, 26, 35, 36, 42, 43, 44, 45] met the inclusion criteria, with one additional study included for descriptive reporting, as shown in the PRISMA flow diagram (Figure 1).

### 3.2 Characteristics of Included Studies and Underlying Causes

This SRMA included eight primary studies [9, 26, 35, 36, 42, 43, 44, 45] to estimate the relapse burden of SAM post‐recovery, with one additional article for descriptive reporting. The included articles represented five regions: four from Amhara [9, 36, 45, 46], two from SNNP [26, 44], one from Oromia [42], one from Benishangul [43], and one from Gambella [35]. Sample sizes ranged from 208 in Gambella [35] to 1273 in Amhara [9]. Relapse rates varied from 10.3% [35] to 34.9% [9]. Four studies [26, 43, 44, 45] employed a cohort design in health facilities, while the remaining five were community‐based [9, 35, 36, 42, 46].

Regarding child age, two studies [26, 44] included children aged 0–59 months, while the remaining seven focused on children aged 6–59 months [9, 35, 36, 42, 43, 45, 46]. Major factors attributed to SAM relapse included rural residence [43, 44], diarrhea and lack of latrine [26, 36], comorbidities such as HIV [26, 43], child age [26, 36], and household food insecurity [9, 36, 42]. Additionally, lack of maternal education and nutritional counseling during recovery [35, 45] significantly contributed to readmission/relapse. In total, 4878 SAM‐treated children were screened, and 958 relapsed cases were reported between November 10, 2017 [9] and May 14, 2021 [36]. The male‐to‐female ratio was 2429 (47.2%) males versus 2723 (52.8%) females, as described in Table 1.

**Table 1:** Characteristics of the included studies and their final treatment outcomes in Ethiopia.

First Author	Publication year	Region	Study setting	Design	Male/Female	Sample size	Relapsed cases	Quality
Abera Labmebeb [26]	2021	SNNR	Health facility	Cohort	352/374	726	86	9
Mohammed Aliye [42]	2023	Oromia	Community	Cross‐sectional	108/106	213	77	8
Endalkachew Birhanu [35]	2023	Gambella	Community	Cross‐sectional	106/102	208	21	9
Fassikaw Kebeded [43]	2022	Benishangul	Health facility	Cohort	333/427	760	82	9
Abera Labmebeb [26]	2021	SNNR	Health facility	Cohort	435/465	900	87	9
Fassikaw and Yimere [45]	2024	Amhara	Health facility	Cohort	242/238	480	48	9
Yibeltal Asmam [36]	2024	Amhara	Community	Cross‐sectional	173/145	318	112	8
Dereje Birhanu [9]	2020	Amhara	Community	Cross‐sectional	680/590	1,273	445	7

### 3.3 The Pooled Relapse Burden of SAM Among Under‐Five Children

**Figure 2:**
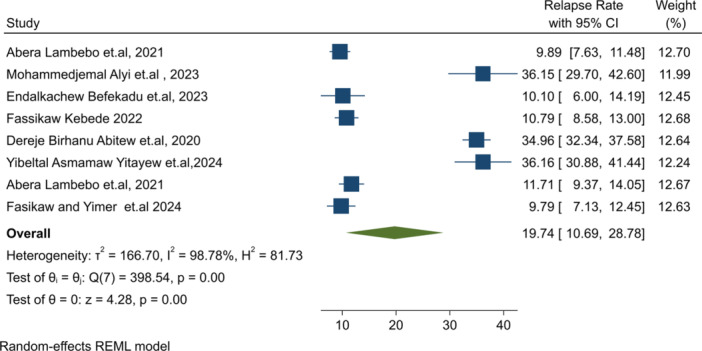
Forest plot for national level pooled relapse burden of SAM post recovery in Ethiopia.

The pooled national estimate for SAM relapse burden after treatment completion was 19.4% (95% CI: 10.69–28.78), with significant heterogeneity among studies (I² = 98.78%, *p* = 0.001), as shown in Figure 2. A leave‐one‐out sensitivity analysis indicated that studies by Mohammed Aliye et al. [42] and Yibeltal et al. [36] had a significant impact on the overall estimate. Removing these studies yielded pooled relapse burdens of 17.49% [42] and 14.49% [36], respectively.

### 3.4 Aggregated Outcome of Included Studies Using SPHERE Indicators

**Figure 3:**
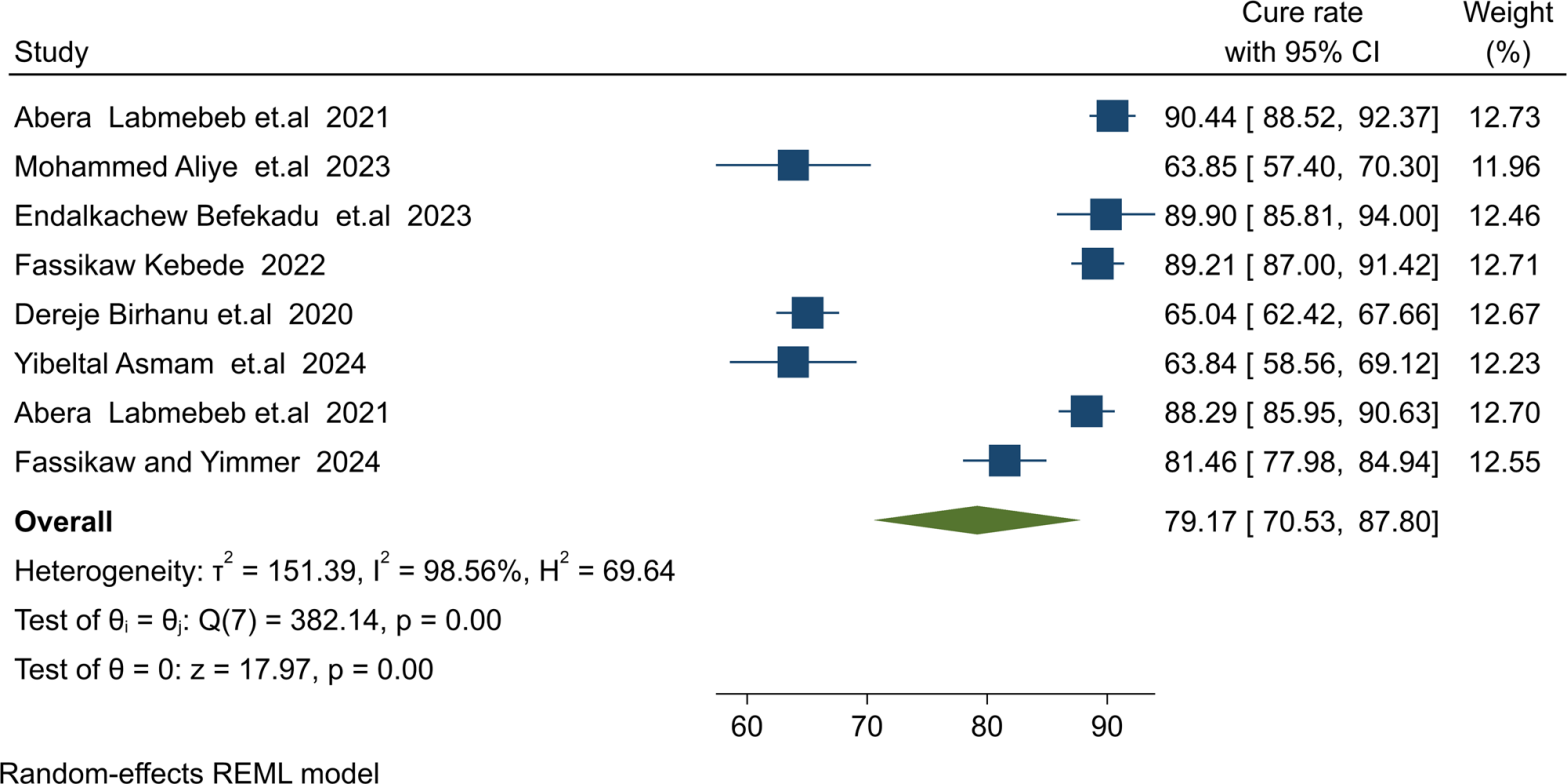
Forest plot for pooled treatment cure rate among result reported studies.

**Figure 4:**
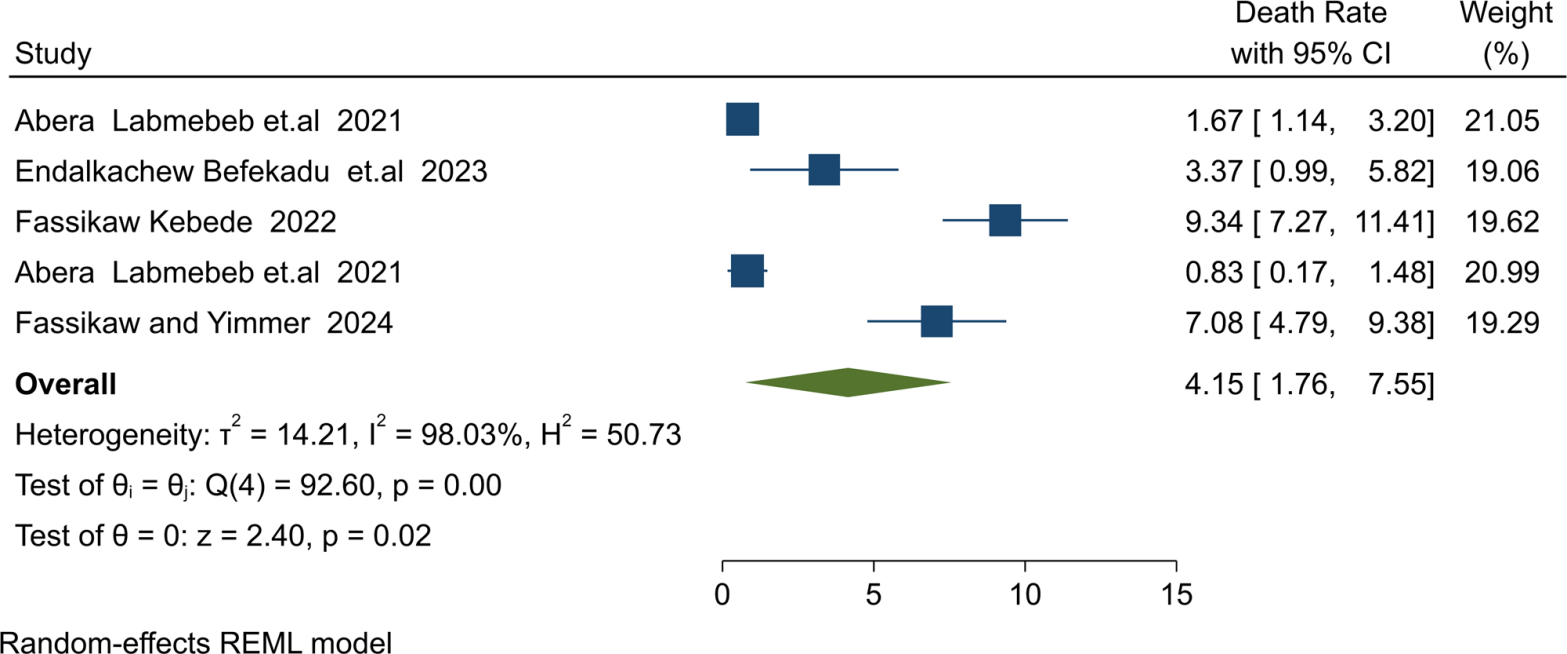
Forest plot for pooled mortality rate estimation among result reported studies.

**Figure 5:**
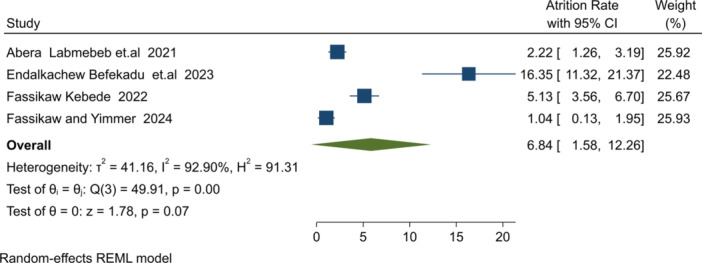
Forest plot for overall attrition rate estimation among result reported studies.

1. **Treatment recovery status**: Eight studies reported SAM treatment outcomes [9, 26, 35, 36, 42, 43, 44, 45]. Most findings were within acceptable SPHERE ranges, though recovery rates in studies by Yibeltal et al. [36] and Dereje et al. [9] were below the reference threshold. The pooled recovery rate was 79.17% (I² = 98.5%, *p* = 0.001), which is within the acceptable SPHERE range (≥ 75%) [3], as shown in Figure 3.
2. **Mortality burden**: Five studies reported mortality during SAM treatment [26, 35, 43, 44, 45]. Most findings were consistent with the SPHERE reference ( ≤ 10%) [3, 47], though two studies from Benishangul Gumuz [43] and Amhara [45] exceeded this threshold. The pooled mortality burden was 4.15% (I² = 98.03%, *p* = 0.001), within the acceptable range (Figure 4).
3. **Attrition rate**: Five studies [26, 35, 43, 44, 45] reported self‐discharge from treatment centers. Three community‐based studies [9, 36, 42] did not report attrition rates. The pooled attrition rate was 5.84% (I² = 98.9%, *p* = 0.001), within the acceptable SPHERE range ( ≤ 10%) [3] (Figure 5).
4. **Relapse burden**: Eight studies [9, 26, 35, 36, 42, 43, 44, 45] reported relapse burden post‐recovery discharge. Relapse rates were highest in Oromia (36.2%) [42] and Amhara (35.2%) [36]. The pooled national relapse burden was 19.4% (I² = 98.78%, *p* = 0.001), which exceeds the SPHERE reference (≤ 5%) [3] (Table 2).

**Table 2:** Performance indicators of included studies compared with SPHERE performance reference.

Research	Total	Recovery rate	Death rate	Attrition rate	Transfer out	Relapse rate	SPHERE project reference values [2, 3]			
							Performance indicators	Overall	Acceptable	Alarming
Abera Labmebeb [44]	760	667 (91.9)	6 (0.8)	20 (2.8)	16 (2.2)	86 (11.8)	Recovery rate	77.9	≥ 75	≤ 50
Mohammed Aliye [42]	213	136 (64.2)	NR	NR	NSR	77 (36.2)	Coverage	50–70	< 70	≥ 70
Endalkachew Befekadu [35]	250	208 (83.2)	7 (2.8)	34 (14.0)	NSR	21 (10.1)	Death rate	12.3	≤ 10	≥ 15
Abera Labmebeb [26]	900	838 (93.1)	6 (0.7)	20 (2.2)	6 (0.7)	86 (9.6)	Length of stay	6.8 WKs	≤ 4 Wks	> 6 WKs
Fassikaw and yimere [45]	480	391 (81.46)	34 (7.0)	5 (1.04)	2 (0.42)	47 (9.79)	Lost to follow‐up	15%	≤ 10	≥ 25
Fassikaw Kebede [43]	721	629 (82.7)	71 (9.1)	39 (5.3)	21 (2.7)	81 (10.8)	Transfer out	4.4%	NS	NS
Yibeltal Asmam [36]	318	206 (65.47)	NR	NR	NR	112 (35.2)	Relapse/readmission	10%	≤ 5%	NS
Dereje Birhanu [9]	1273	828 (65.04)	NR	NR	NR	445 (34.9)	Non response rate	NS	NS	NS
Over all pooled estimation		79.17% (I2 = 98.5%)	4.5%,(I2 = 98.03)	5.84%, (I² = 98%)	3.5% I2 = 97.8%,	19.4% I2 = 98.7%				

Abbreviations: NR, not reported; NS, no standers of referee; WKS, weeks.

### 3.5 Subgroup Analysis and Handling Heterogeneity

**Figure 6:**
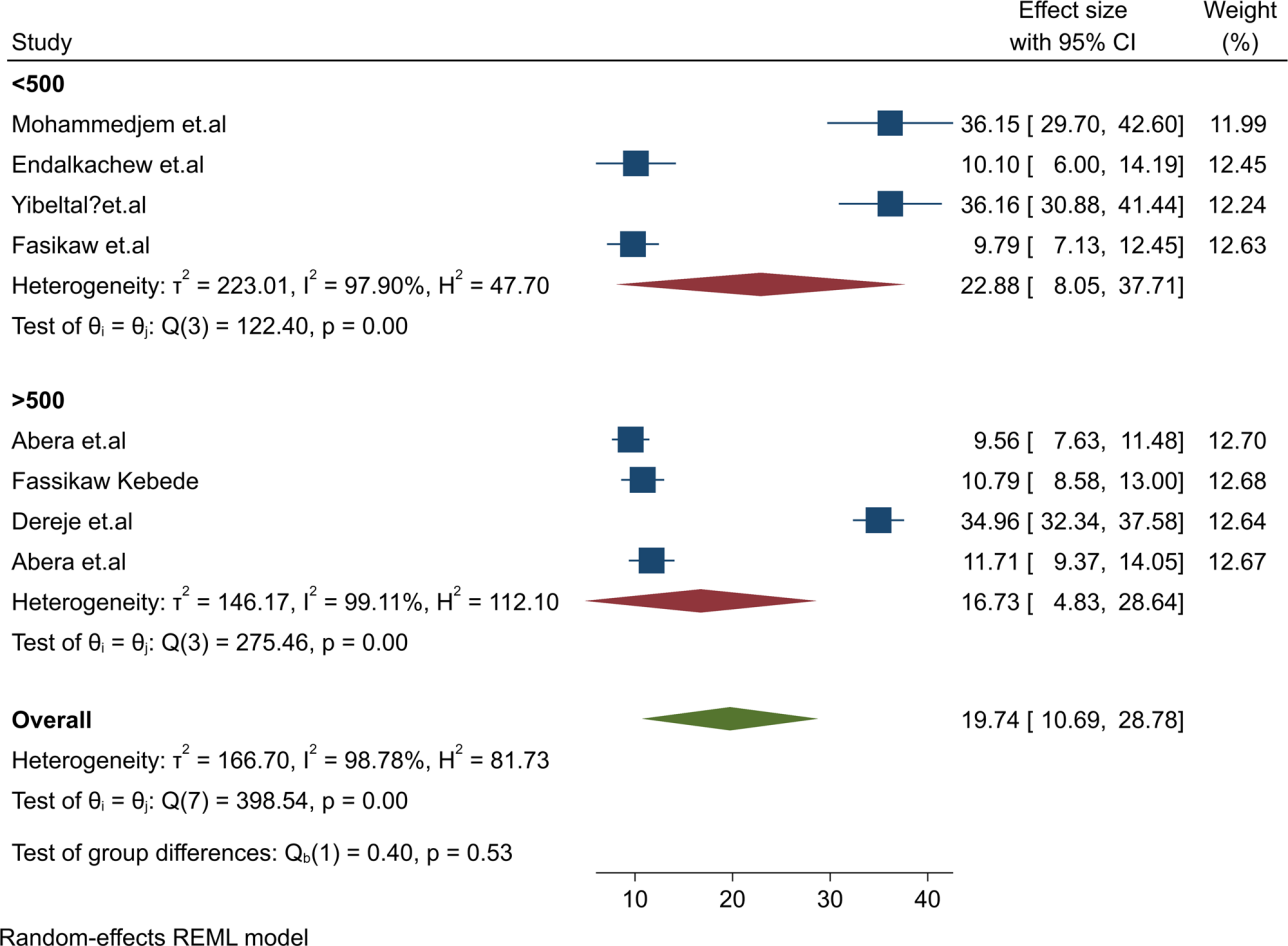
Subgroup analysis by sample size and relapse burden of SAM post treatment discharged.

**Figure 7:**
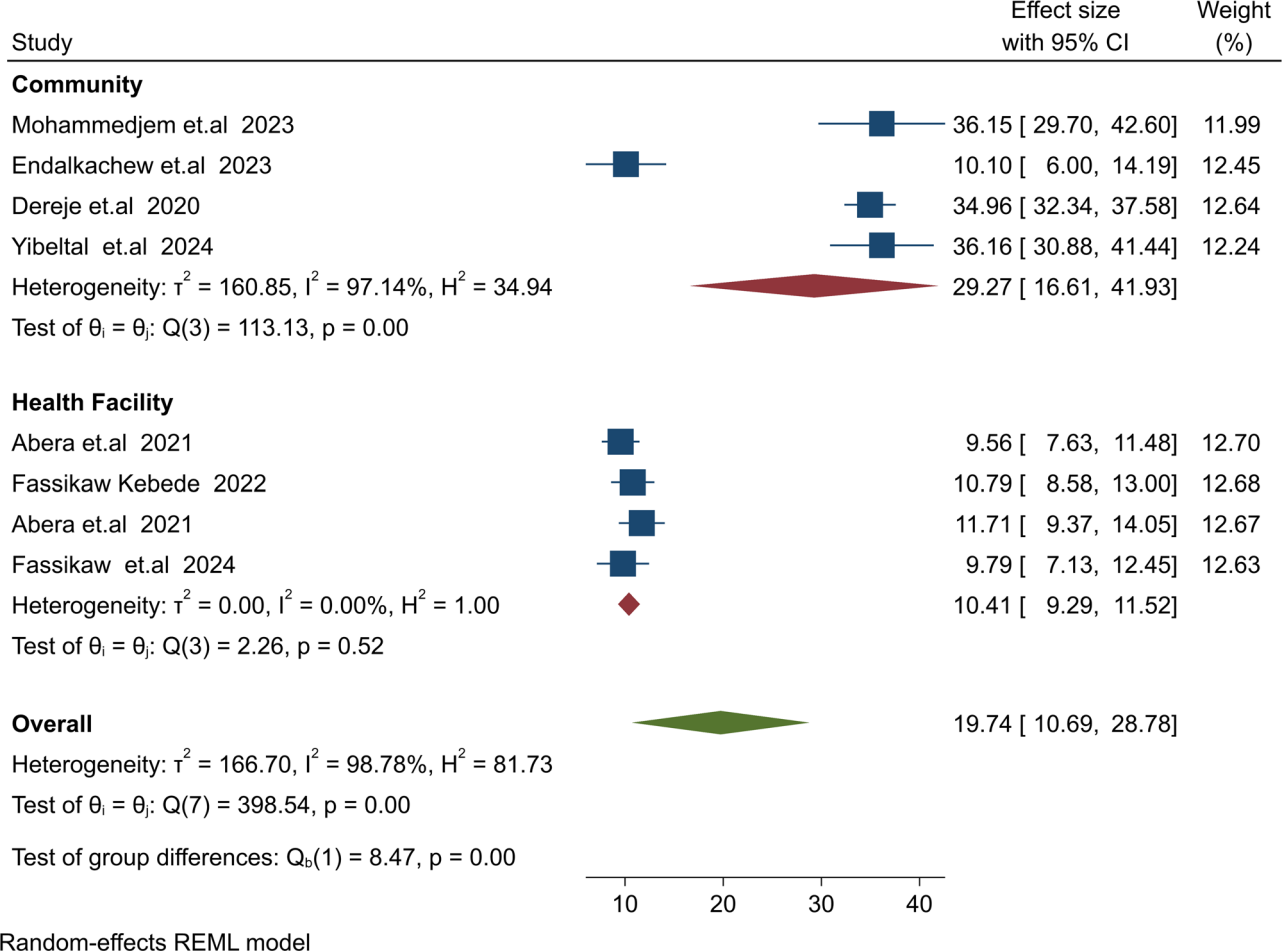
Subgroup analysis by study‐setting and relapse burden of SAM post treatment discharged.

Subgroup analysis was conducted to address significant heterogeneity (I² = 98.78%, *p* = 0.001). Analyzes were stratified by sample size, study setting, follow‐up time, and region, as shown in Table 3. The relapse burden was higher in the Amhara region (RB = 26.9%) compared to the SNNP region (RB = 10.52%). Cross‐sectional studies reported a higher relapse burden (27.37%) than facility‐based cohort studies (10.41%). Studies with sample sizes < 500 had a higher relapse burden (22.88%) compared to those with ≥ 500 participants (16.73%) (Figure 6). Community‐based studies had a higher relapse burden (29.27%) compared to health facility‐based studies (10.41%) (Figure 7).

**Table 3:** Subgroups analysis of relapse Burden of SAM among included studies in Ethiopia.

Studies variables	Categories	Number of studies	Relapse burden	95%CI	I^2^	Estimated *p* value
Sample size	< 500	4	22.88	8.07–37.7	97.9	*p* = 0.001
	≥ 500	4	16.73	4.83–28.6	99.1	*p* = 0.001
Study design	Cohort design	4	10.41	9.29–11.52	98.7	*p* = 0.051
	Cross‐sectional	4	27.37	27.37–44.9	99.6	*p* = 0.001
Study setting	Health facilities	4	29.27	9.29–11.52	97.14	*p* = 0.001
	Community‐based	4	10.41	16.27–41.93	0.001	*p* = 0.52
Study regions	Amhara	3	26.9	9.9–43.8	98.79	*p* = 0.001
	SNNR	2	10.52	8.42–12.62	48.56	*p* = 0.016
	Oromia (Harari)	1	36.1	29.7–42.8	0.11	*p* = 0.001
	Benishangual	1	10.79	8.52–13.1	—	*p* = 0.001
	Gamabela	1	10.10	6.0–14.19	—	*p* = 0.001

### 3.6 Publication Bias and Sensitivity Analysis

**Figure 8:**
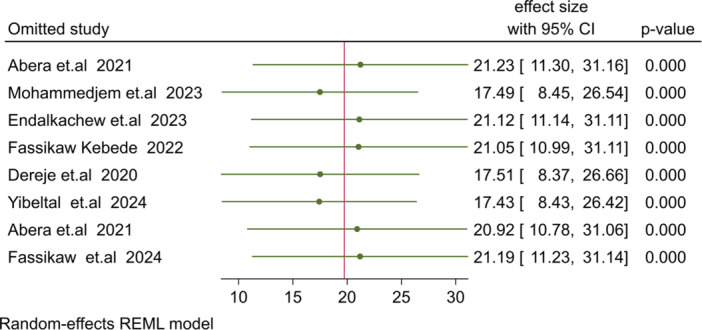
A‐leave‐ out sensitivity analysis for eastimation of relapse burden.

**Figure 9:**
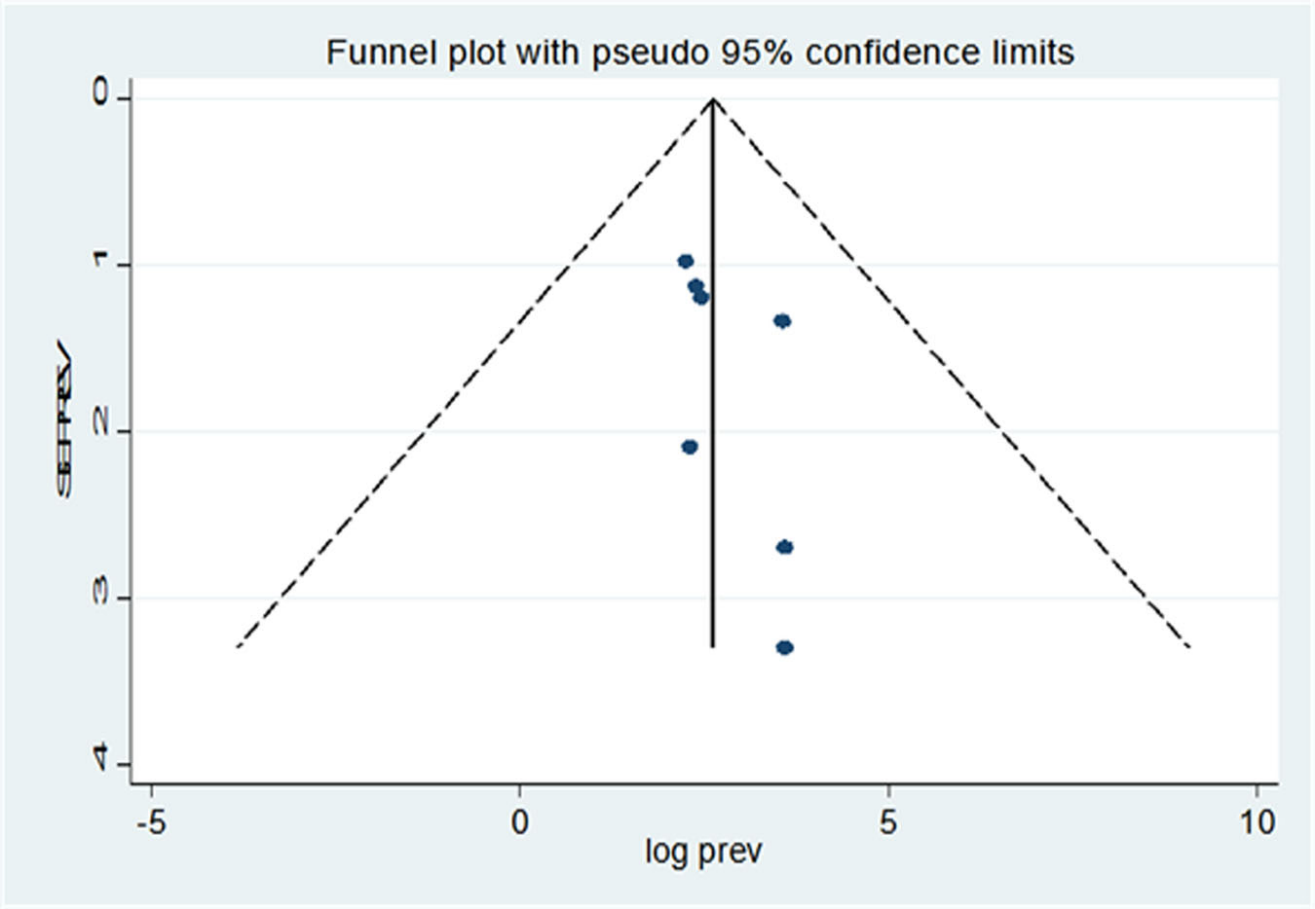
Funnel plot for publication biases assessment among included studies.

Publication bias was assessed using funnel plots and Begg's and Egger's regression tests. The leave‐one‐out sensitivity analysis showed that no single study significantly affected the pooled estimate (Figure 8). The funnel plot indicated no evidence of publication bias, with all studies falling within the plot (Figure 9). Egger's regression test confirmed no significant publication bias (*p* = 0.396) (Table 4).

**Table 4:** Egger's regression test related for relapse burden of SAM post‐recovery in Ethiopia.

Std_Eff	Coeff.	Std. Err	t	P > |t |	(95% Conf. Interval)
Slope	2.297281	0.5271665	4.36	0.002	(1.0816–3.5129)
Bias	0.2796406	0.3115238	0.90	0.396	(−0.4387 to 0.9980)

### 3.7 Risk Factors for Relapse of SAM

**Figure 10:**
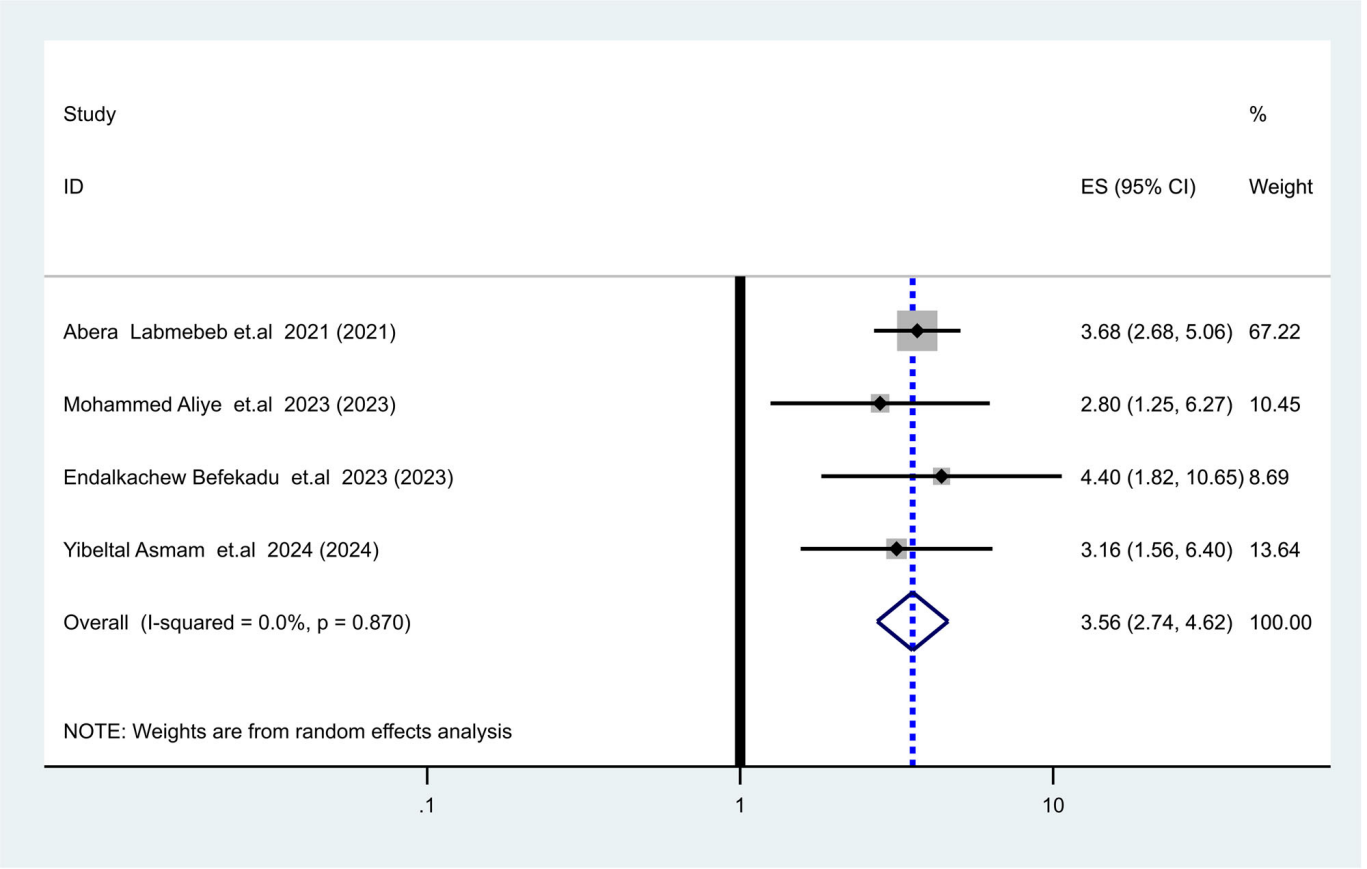
Association between MUAC ≤ 125 mm discharging criteria and relapse of SAM.

Adjusted odds ratios from primary studies were categorized into six themes to identify risk factors for SAM relapse in Ethiopia. Using weighted inverse variance random‐effects meta‐regression, discharge based on MUAC ≥ 125 mm (Pooled HR = 3.6; 95% CI: 2.8–4.6, I² ≤ 15%, *p* = 0.00) was associated with a nearly fourfold increased risk of relapse (Figure 10). Similarly, children from households with food insecurity had a sixfold increased likelihood of relapse (Pooled HR = 6.33; 95% CI: 0.99–42.5, I² = 94.4%, *p* = 0.001) (Table 5).

**Table 5:** Factors associated with relapse burden of SAM for under‐five children in Ethiopia.

Variables	Categories	No. of studies	Reference	Pooled AOR	95% CI	I2%	*p* value
Resident	Rural	2	[43, 45]	2.7	0.39–18.42	0.55	*p* = 0.07
Edema at first admission	Yes	3	[26, 43, 44]	1.8	1.39–11.2	97.5	*p* = 0.111
MUAC ≥ 125 mm for discharge	≥ 125 cm	4	[26, 35, 36, 42]	3.6	2.8–4.6	0.01	*p* = 0.001*
HH food and wealth status	In secured	3	[9, 36, 42]	6.33	3.97–42.5	94.4	0 = 0.001*

## 4 Discussion

This study assessed the relapse burden of SAM post‐treatment recovery across five Ethiopian regions. It estimated the aggregated relapse burden, recovery outcomes, mortality, and attrition rates using SPHERE performance indicators among 4878 children discharged as recovered. The pooled relapse burden in Ethiopia was 19.4% (95% CI: 10.69 to 28.78). This is lower than previous reports: 41% in Malawi [23], 26.1% in Mali [14], 27.5% in a global systematic review [48], and 44.2% in Congo [49]. These discrepancies may be due to variations in study populations, periods, and definitions of relapse. For instance, in Mali, a prospective cohort study of 420 children discharged using MUAC ≤ 125 mm reported a 26% relapse rate, increasing to 27% when MUAC was < 125 mm within 1 year of follow‐up [14]. In Burkina Faso, relapse was defined as WHZ < −2 Z‐score and MUAC < 12 cm [15], whereas in Ethiopia, relapse was defined as WHZ between −2 and −3 or MUAC between 115 mm and 125 mm [16], potentially increasing readmission cases.

Conversely, the relapse rate in this study was higher than systematic review findings of 8% in Niger [48], 15.4% in Burkina Faso [15], and 17% in the USA [19]. Differences may be influenced by distance to healthcare and household size. For example, caregivers with more than two children often face heightened stress and conflicting priorities, leading to self‐discharge from treatment centers [26, 27, 28]. In Benishangul Gumuz [50], a retrospective cohort study of 760 children found that 9.7% self‐discharged.

Aggregated results for recovery transfer out, and attrition rates were consistent with SPHERE standards [9, 36]. However, the transfer‐out rate (TOR = 3.5%) and relapse rate (RR = 19.4%) did not align with national minimum criteria (≤ 15% and ≤ 5%, respectively). This may be due to persistent regional conflicts; in Tigray, 12.5% of under‐five deaths were linked to malnutrition, and in Amhara, 44.42% of displaced children developed SAM [29, 51]. Delayed case presentation, poor adherence to treatment guidelines, and premature discharge also increase readmission rates [16].

Regarding predictors, household food insecurity significantly increased the risk of relapse, consistent with studies in North Africa [18] and LMICs [52]. This may be due to a lack of post‐discharge nutritional counseling. A cluster‐randomized trial showed that recovery‐focused nutritional counseling improved sustained recovery post‐discharge [53]. Similarly, a study in rural Angola found that counseled children had higher weight gain (9.3 g/kg/day) [54].

Discharge based on MUAC ≥ 125 mm was associated with a fourfold increased risk of relapse [18, 49], consistent with findings in Nepal [55], Mali [14], and a multi‐country study (Mali, Somalia, South Sudan) [24]. In Mali, children discharged with MUAC ≤ 130 mm had a 26.1% relapse rate within 6 months [14], and discharge with MUAC ≥ 125 mm tripled the risk of readmission [56].

### 4.1 Strengths and Limitations of the Study

This is the first meta‐analysis on SAM relapse in Ethiopia, providing valuable insights for stakeholders and treatment institutions. Strengths include the inclusion of all observational designs, which better identify cause‐effect relationships and reduce methodological variability. All SAM treatment outcomes were assessed using SPHERE performance indicators. Limitations include the exclusion of qualitative studies and non‐English articles, a limited number of included studies, lack of representation from all Ethiopian regions, and small sample sizes in some studies, which may affect the pooled estimates.

## 5 Conclusion and Recommendation

In this SRMA, nearly one in five children under five was readmitted for SAM re‐treatment post‐recovery. Relapse burden and self‐discharge rates exceeded national SPHERE performance indicators. Therefore, the use of MUAC indices as discharge criteria should be reconsidered and re‐standardized. Comprehensive nutrition education, household food aid, and continued follow‐up are strongly recommended for children treated and declared cured of SAM.

## 6 Implications and Contribution

This study enhances understanding of the need for early tailored interventions, including post‐recovery follow‐up and relevant policy changes to address the relapse burden of SAM. It contributes significantly to understanding SAM relapse in Ethiopia, offering critical insights for improving treatment outcomes and reducing malnutrition burden. By identifying key predictors and regional disparities, it provides a foundation for targeted interventions and policy reforms. The findings also highlight the need for further research to address methodological heterogeneity and improve primary study quality.

## Author Contributions

**Fassikaw Kebede Bizuneh:** conceptualization, investigation, funding acquisition, writing – original draft, methodology, validation, visualization, writing – review and editing, software. **Birtukan Gizachew:** conceptualization, methodology, validation, visualization, writing – review and editing, supervision, data curation, formal analysis. **Tsehay Kebede:** conceptualization, funding acquisition, writing – original draft, methodology, validation, visualization, writing – review and editing, project administration, formal analysis, software. **Abebe Fenta:** investigation, funding acquisition, writing – original draft, visualization, validation, methodology, formal analysis, project administration, resources, supervision. **Desalegn Nazi:** writing – original draft, writing – review and editing, visualization, validation, methodology, project administration, data curation. **Sefineh Fenta:** conceptualization, methodology, software, data curation, project administration, visualization, funding acquisition, writing – review and editing. **Worku Misganaw:** investigation, conceptualization, validation, formal analysis, supervision, data curation, writing – original draft, project administration, visualization, writing – review and editing.

## Ethics Statement

The authors have nothing to report.

## Conflicts of Interest

The authors declare no conflicts of interest.

## Transparency Statement

The lead author Fassikaw Kebede Bizuneh affirms that this manuscript is an honest, accurate, and transparent account of the study being reported; that no important aspects of the study have been omitted; and that any discrepancies from the study as planned (and, if relevant, registered) have been explained.

## Data Availability

The data that supports the findings of this study are available in the supplementary material of this article.
